# Automatic and objective oral cancer diagnosis by Raman spectroscopic detection of keratin with multivariate curve resolution analysis

**DOI:** 10.1038/srep20097

**Published:** 2016-01-25

**Authors:** Po-Hsiung Chen, Rintaro Shimada, Sohshi Yabumoto, Hajime Okajima, Masahiro Ando, Chiou-Tzu Chang, Li-Tzu Lee, Yong-Kie Wong, Arthur Chiou, Hiro-o Hamaguchi

**Affiliations:** 1 Institute of Biophotonics, National Yang-Ming University, No. 155, Sec. 2, Li-nong Street, Taipei, Taiwan; 2 Department of Applied Chemistry and Institute of Molecular Science, National Chiao Tung University, 1001 Ta Hsueh Road, Hsinchu, Taiwan; 3 Research Organization for Nano & Life Innovation, Waseda University, 513 Wasedatsurumaki-cho, Shinjuku, Tokyo, 162-0041 Japan; 4 Department of Dentistry, Taichung Veterans General Hospital, No. 1650, Sec. 4 Taiwan Boulevard, Xitun Dist., Taichung, Taiwan; 5 Biophotonics & Molecular Imaging Research Center (BMIRC), No. 155, Sec. 2, Li-nong Street, Taipei, Taiwan

## Abstract

We have developed an automatic and objective method for detecting human oral squamous cell carcinoma (OSCC) tissues with Raman microspectroscopy. We measure 196 independent Raman spectra from 196 different points of one oral tissue sample and globally analyze these spectra using a Multivariate Curve Resolution (MCR) analysis. Discrimination of OSCC tissues is automatically and objectively made by spectral matching comparison of the MCR decomposed Raman spectra and the standard Raman spectrum of keratin, a well-established molecular marker of OSCC. We use a total of 24 tissue samples, 10 OSCC and 10 normal tissues from the same 10 patients, 3 OSCC and 1 normal tissues from different patients. Following the newly developed protocol presented here, we have been able to detect OSCC tissues with 77 to 92% sensitivity (depending on how to define positivity) and 100% specificity. The present approach lends itself to a reliable clinical diagnosis of OSCC substantiated by the “molecular fingerprint” of keratin.

Molecular-level tissue characterization is highly potent for cancer diagnosis. As a tissue starts becoming cancerous, specific biomolecules are overexpressed or aberrantly expressed, which can be used as cancer molecular markers. If we can detect these molecular markers spectroscopically, it would lead to a new molecular-level cancer diagnosis with high objectivity.

Keratin-family proteins (M.W. 40000 ~ 67000) are major components of fibrous structural proteins in epithelial cells. They play important roles in the formation of cytoskeleton network and help maintain the structural integrity of cellular morphology^1^,^2^. Several studies have shown that keratin is aberrantly expressed in many different types of human epithelial cancers including skin cancer, lung cancer, breast cancer, cervix cancer, esophagus cancer, salivary gland cancer and oral cancer^3^,^4^,^5^,^6^. In the present study, we focus on oral cancer. Oral squamous cell carcinoma (OSCC) is one of the most common cancers (95% in oral malignancy) in oral cavity. Keratin is a well-established molecular marker of OSCC; oral malignancy can be diagnosed by detecting the variations in keratin expression between OSCC and normal oral tissues^7^,^8^. At present, keratin in oral tissues is detected and analyzed by means of immunohistochemistry (IHC). However, IHC is expensive, time-consuming and needs specialist attention. An economic and straightforward alternative for keratin detection in oral tissues is longed for.

Spectroscopic methods for cancer diagnosis have made a rapid progress in recent years. In particular, Raman spectroscopy has been proven to be effective for discriminating cancerous against normal oral tissues^9^,^10^,^11^,^12^,^13^,^14^,^15^,^16^,^17^,^18^,^19^. Spectroscopic discriminations of cancer tissues in these previous studies are mostly based on Principal Component Analysis (PCA) in conjunction with statistical multi-parameter analyses. The key advantage of PCA is that once a spectral data set obtained from tissues is analyzed and separated into several particular categories, then a new spectrum can automatically be assigned to one of those categories, for example, cancerous vs. normal. However, PCA does not extract detailed molecular spectral information from the categorized spectra and its physical basis of categorization tends to remain unclear.

Biological tissues are highly heterogeneous and their Raman spectra vary widely depending on the position where they are measured. Furthermore, molecular compositions of tissues are so complicated that their raw Raman spectra can hardly be interpreted. In order to accomplish global tissue analysis effective for cancer diagnosis, we need to (1) collect Raman spectra from as many as possible points from a tissue sample, (2) estimate the number of principal spectral components contained in this large number of Raman spectra, (3) decompose the raw spectra into spectrally interpretable components and finally (4) objectively characterize tissues according to the extracted spectral information. The methodology employed up to now relies greatly on specialized “spectroscopic eyes”, which has not facilitated its practical applications in cancer diagnosis. The aim of the present study is to develop an automatic and objective method for discriminating oral cancer tissue by detecting keratin without any specialized knowledge of spectroscopy. We (1) collected a total of 196 Raman spectra from one oral tissue sample, (2) estimated the number of principal spectral components by Singular Value Decomposition (SVD), (3) applied Multivariate Curve Resolution-Alternating Least Square (MCR-ALS)^20^,^21^ analysis to decompose a large set of complicated spectra into spectrally interpretable components and (4) carried out the spectral matching analysis between these MCR-decomposed spectral components and the keratin standard spectrum, to objectively discriminate OSCC against normal tissues via Unit Normalized Euclidean Distance (UNED).

The present method fully utilizes the Raman spectral information (molecular fingerprint) of the marker molecule, keratin; in contrast, in PCA approaches, Raman spectra are treated just as two-dimensional signature for a pattern recognition analysis without referring much to their physicochemical meanings. The identification of keratin signature is automatically and objectively achieved with the use of UNED, making the whole analysis readily acceptable for non-specialists of spectroscopy.

## Results

### Determination of the number of principal spectral components contained in the observed spectra

**Figure 1:**
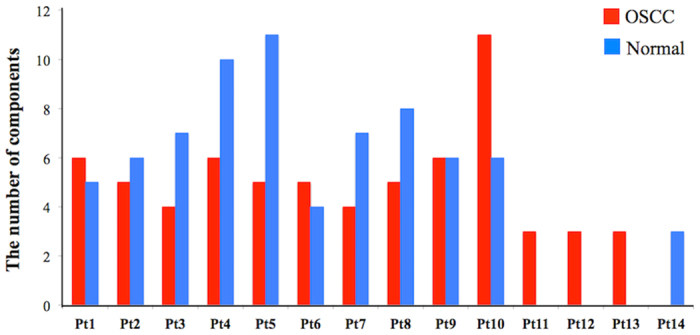
The number of principal spectral components *k* in 14 patients’ OSCC and normal oral tissues with signal-to-noise ratio (S/N) higher than 4. Different *k* values show the variation of samples obtained from different patients.

We first determined the number of principal spectral components, *k*, based on the signal-to-noise ratio (S/N) consideration described in the Methods Section. We have tried several threshold values and finally set it to S/N = 4. If the threshold value is higher, we have fewer spectral components (smaller *k* values) in which keratin signatures are likely to be mixed up with other protein signatures. If the threshold value is lower, we have more spectral components (larger *k*) in which keratin signatures are likely to be contaminated with noise and dispersed among plural spectral components. The present threshold value, S/N = 4, is the optimized value for the present data set of 196 × 24 = 4704 Raman spectra from 14 patients. This threshold is to be further optimized with larger number of data from larger number of patients in the future. For present, we used the threshold value, S/N = 4, to show that the following automatic analysis proceeds successfully, once the threshold value is fixed. The determined *k* values are shown in Fig. 1 for Patient-1 ~ Patient-10 (OSCC and normal tissue samples), Patient-11 ~ Patient-13 (OSCC) and Patient-14 (normal). These different *k* values show the variation of samples obtained from different patients.

### MCR-ALS fitting to decompose the observed spectra into spectrally interpretable components

**Figure 2:**
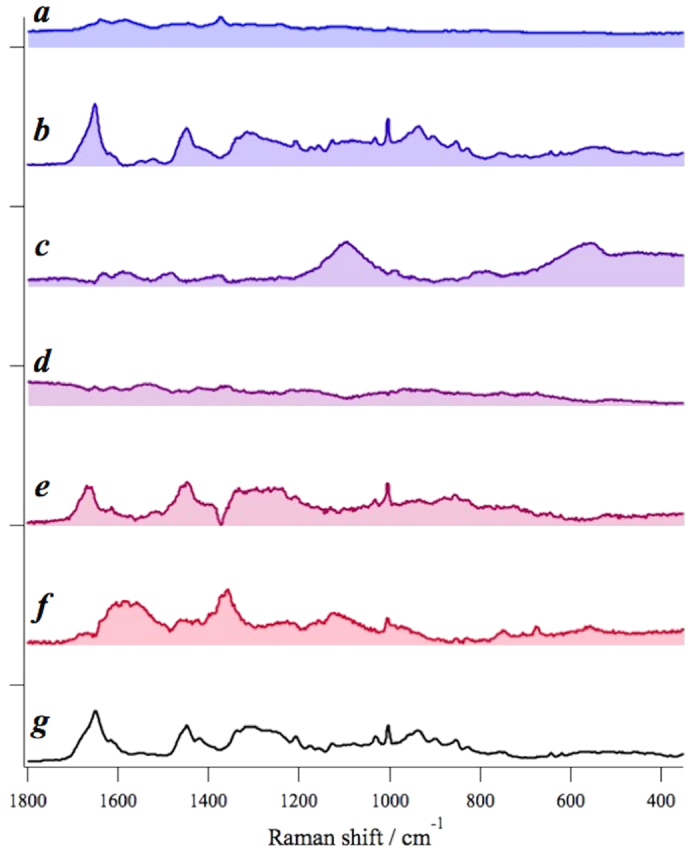
MCR-ALS spectral components from Patient-1 OSCC tissue sample (**a**–**f**) and the standard keratin spectrum (**g**); the spectral component (**b**) shows excellent correspondence with the standard keratin spectrum (**g**).

After determining the number of principal spectral components in tissue, we applied MCR-ALS analysis (see Methods) to decompose the complicated raw spectra into spectrally interpretable components. The MCR spectral components of the OSCC tissue of Patient 1 is shown in Fig. 2(a–f). The normalized residual R_ij_ = |(A_ij_-WH_ij_)/A_ij_| at the *i-th* row and the *j-th* column is less than 5 ~ 7%, indicating that the principal signatures in the original data set *A* are well represented by the product *WH* with MCR decomposition. Thus obtained MCR decomposed spectra are readily compared with the standard keratin spectrum in Fig. 2g. We notice that one of the MCR components, Fig. 2b, shows excellent correspondence with the standard keratin spectrum with characteristic protein peaks at 1650 *cm*^*−1*^ (Amide I), 1450 *cm*^*−1*^ (CH bend), 1200 ~ 1350 *cm*^*−1*^ (Amide III), 1003 *cm*^*−1*^, 1030 *cm*^*−1*^ (Phenylalanine), 937 *cm*^*−1*^, 890 *cm*^*−1*^ (C-C stretch), 850 *cm*^*−1*^ and 830 *cm*^*−1*^ (Tyrosine), especially with a broad band feature around 1200 ~ 1350 *cm*^*−1*^. The spectral components Fig. 2a,d are ascribable to autofluorescence. Raman spectrum of glass substrate is separated out as Fig. 2c. Proteins other than keratin seem to be included in components Fig. 2e and hemoglobin is likely to be contained in Fig. 2f^22^.

**Figure 3:**
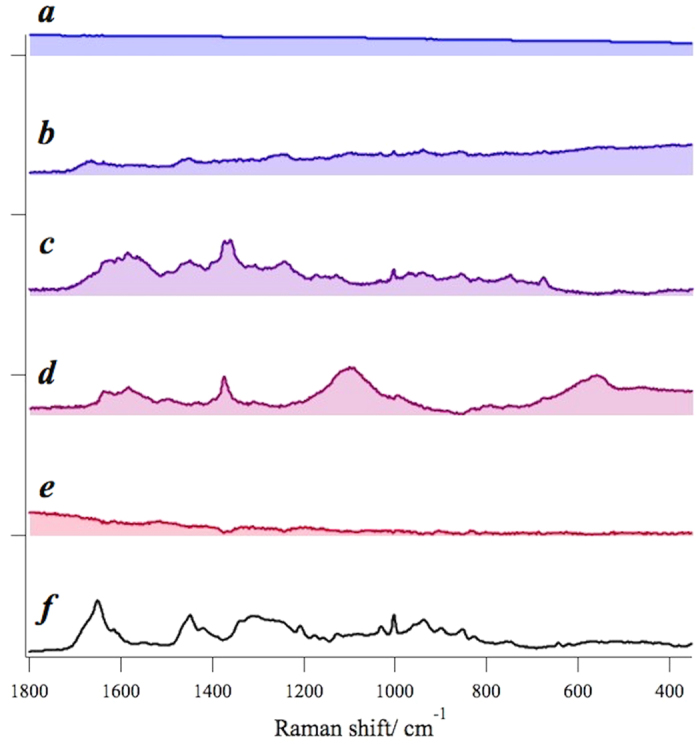
MCR-ALS spectral components from Patient-1 normal tissue sample (**a**–**e**) and the standard keratin spectrum (**f**). No MCR spectral components seem to match the standard keratin spectrum.

The result for the normal oral tissue of Patient 1 is shown in Fig. 3(a–e). The normalized residual is less than 6%. The keratin spectrum does not seem to match any spectral components from the normal oral tissue. Spectral components in Fig. 3a,b and e are ascribed to autofluorescence. The spectral component in Fig. 3c is likely to contain protein signatures with a characteristic band of phenylalanine residue at 1003 *cm*^*−1*^. Prominent signatures in Fig. 3d are from glass substrate.

Although we discuss the assignments of the decomposed components, we can process the MCR and the spectral matching evaluation (next step) analysis without them. The process is fully automatic and does not require spectral assignments in our protocol.

### Spectral matching between principal MCR spectral components and the standard spectrum of keratin

By the preceding MCR analysis, we obtained decomposed spectral components. In order to evaluate how “similar” these spectral components are to the standard keratin spectrum, without relying on specialized “spectroscopic eyes”, we employed the idea of spectral matching. We have tried several indicators of spectral matching including Spectral Angle (SA), Euclidean Distance (ED) and Spectral Information Divergence (SID)^23^,^24^,^25^ and found that Unit Normalized Euclidean Distance (UNED) (described in Methods) provides the clearest measure of the spectral similarity.

**Figure 4:**
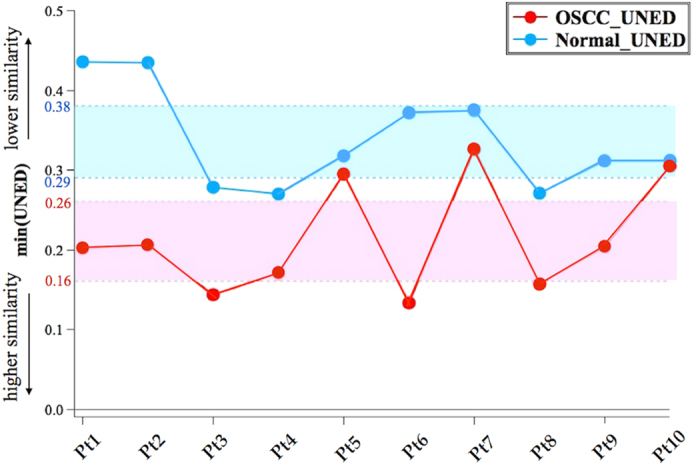
UNED results of ten paired-patients (including OSCC and normal tissues) with the confidence intervals of OSCC (UNED = 0.16 ~ 0.26) and normal (UNED = 0.29 ~ 0.38). The upper bound of OSCC confidence interval (UNED = 0.26) can separate OSCC and normal tissues effectively.

We calculated UNEDs between each MCR-decomposed spectrum and the standard keratin spectrum in the region 800 ~ 1800 *cm*^*−1*^. We then picked up the minimum UNED value among decomposed spectra in each tissue sample to quantify the “highest similarity” of each sample. We first used the ten paired-samples (OSCC/normal) from the same ten patients for comparison. The result is shown in Fig. 4 with ten red points for OSCC and ten blue points for normal tissue samples, respectively.

We note that OSCC points tend to have smaller UNED values than the corresponding normal points. This trend indicates that the decomposed spectral components in the OSCC tissue samples have higher similarity with the standard keratin spectrum than those in the normal. The 95% confidence interval of the OSCC points is 0.16 < UNED < 0.26, while that of the normal points is 0.29 < UNED < 0.38. These two 95% confidence intervals do not overlap with each other, showing that UNED clearly distinguishes OSCC and normal oral tissues with high accuracy and specificity. If we simply take the upper bound of OSCC confidence interval (UNED = 0.26) as the threshold value, we can separate OSCC and normal groups with 70% accuracy in OSCC tissues (failure for Patient 5, 7, 10) and 100% specificity in normal tissues. The three false points, which were histologically diagnosed as cancerous, may well correspond to the preliminary stage of cancer that has more cancerous tissues than normal (see discussion below).

With the threshold UNED = 0.26, we analyzed the other three independent OSCC and one independent normal tissue samples. The three OSCC tissue samples show the UNED similarity values of 0.22, 0.13 and 0.22, respectively. These values are all smaller than 0.26. The normal tissue sample shows the value 0.38, which is much higher than 0.26. If we include the three independent OSCC samples, the accuracy increases from 70% to 77%. 100% specificity does not change if we add one normal sample in the analysis.

## Discussion

In the present study, we have developed an automatic and objective method for oral cancer diagnosis by applying the MCR-ALS analysis with spectral matching. In spectral matching, we compared the distance, UNED, between normalized MCR-decomposed spectral components and the normalized standard keratin spectrum to evaluate their “similarity”. The UNED “similarity” value tells us how much the MCR-decomposed spectra contain the characteristic Raman signature of keratin. When UNED value is small, the decomposed spectral component contains much signature of keratin. When UNED value is large, the decomposed spectral component contains less keratin signature. Our results indicated that, from the OSCC tissue samples, high similarity was always found for one of the decomposed spectral components but that no spectral component showed high similarity from the normal tissue samples. Keratin signature was successfully captured from the OSCC tissue samples but not from the normal. Keratin in the normal tissue samples was not detected by MCR primarily because of the much less keratin amount in normal tissues than in OSCC^3^. In addition, spatial distribution of keratin may also play certain roles in the MCR decomposition. Note that the MCR decomposition is based on the differences not only in the spectral profile but also in the spatial distribution. It is likely that the keratin spatial distribution in OSCC is different from that in normal tissues. OSCC tissues may consist of a homogenous population of cells at one particular stage of differentiation, whereas normal tissues consist of cells at different stages of differentiation^26^,^27^. Cancer cells in OSCC tissues are likely to have more chance to stay at G_2_ phase with aberrant keratin syntheses and produce localized spatial distribution of keratin. On the contrary, normal cells mostly progress at different stages of differentiation, M, G_1_ and G_2_ phases, to have keratin randomly distributed spatially. In the present study, we randomly measured the points in tissue samples to obtain global spectral information. We anticipate that specific areas of tissues can be examined by this approach for comparison with immunohistochemical staining result, and use the spatial distribution of keratin, *H*_*i*_ in Equation 4, to further substantiate the discrimination of OSCC tissues against the normal tissues.

In spectral matching, the threshold UNED = 0.26 was set to discriminate OSCC against normal oral tissues from the same patient. The UNED value could also help elucidating the metastasis condition of cancer. The marginal region (UNED = 0.26 ~ 0.29 in Fig. 4) probably represents metastatic cancer cells gradually accessing into normal tissues. With UNED = 0.26 discrimination, the UNED values were larger than 0.26 for the three false points in Patients −5, −7, −10; hence, these were not identified as cancerous. However, their UNED values were smaller than the corresponding values of the normal. If we make comparisons of the UNED values within the same patient, the OSCC tissue samples always show lower UNED values than the normal (for Patient-10, they almost overlap). In that sense, the two OSCC tissue samples, Patients −5, −7, can probably be diagnosed as suspicious (though not identified as cancerous) for having more cancerous tissues than normal. If we regard these suspicious samples as positive, the accuracy increases from 77% to 92%. The pair comparison of the UNED values may provide further information on the metastasis condition of oral cancer.

## Methods

### Tissue samples

Use of tissue samples was approved by Institutional Review Board of the Taichung Veterans General Hospital. All experiments were performed in accordance with the approved guidelines and regulations. Informed consent was obtained from all subjects. Samples from fourteen oral cancer patients included ten paired (cancer and normal tissue samples from the same patient), three independent cancer and one independent normal tissue samples. Cancerous oral tissue samples were histologically confirmed as oral squamous cell carcinoma (OSCC). All tissue samples, immediately after surgical removal, were flash-frozen at −196 degrees Celsius and stored in liquid nitrogen. Tissue samples once stored at liquid nitrogen temperature were then embedded in optimum cutting temperature (OCT) compound and sectioned in a microtome in approximately ten-micrometer thick and were mounted on glass slides. Standard keratin sample was prepared from human stratum corneum, which was known to contain 80% keratin^28^. Practically, it was obtained from stratum corneum cut out from the heel of one of the authors. The sample was soaked in a 1:1 mixture of methanol and chloroform overnight and then was immersed in deionized water^29^. The standard keratin sample that we have taken from human stratum corneum is extensively used as the standard antigen in immunostaining detection of keratin in squamous cell carcinoma (SCC) tissues^3^.

### Raman microspectroscopy

We used a laboratory-constructed Raman microspectrometer for all the Raman measurements. The 488 *nm* line of an Ar-ion laser (CVI Melles Griot) was used for excitation with a power of about 1 *mW* at the sample point. The laser beam was focused into the sample by using a non-immersion 40X,

NA = 0.6, objective (Olympus, LUCPlanFL N). The laser spot size at the sample was estimated to be about 1 *μm*. The back-scattered light was collected by the same objective lens and was focused on to the entrance slit of a polychromator (Andor, SR303i-BNS). A 1200-*grooves/mm* grating was used to disperse scattered light. The signal was detected by a CCD detector (Andor, DU401A-BV) cooled to −80 °*C*. The acquisition time was 60 *sec* for each measurement. The Raman spectrum of indene was acquired for wavenumber calibration^30^. The Raman spectra of all samples were recorded in the 300–2000 *cm*^*−1*^ wavenumber region, which covers most Raman signatures observed from oral tissues.

**Figure 5:**
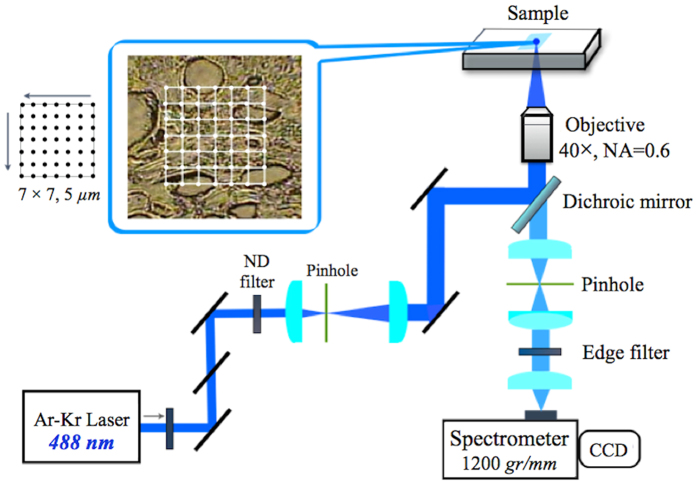
A schematic diagram of the laboratory-constructed Raman microspectrometer.

In the present study, we emphasized on extracting global molecular information of tissues. Therefore, we tried to globally and randomly measure as many points as possible without specific localization in a tissue sample. A piezo X-Y stage (Physilk Instrumente) was used to scan 7 × 7 = 49 points in one region of a tissue sample, with the distance of 5 *μm* between two adjacent points (Fig. 5). The same measurement was repeated four times at different regions of the sample and a total of 196 spectra were collected for each sample for subsequent analysis.

### Data Analysis

**Figure 6:**
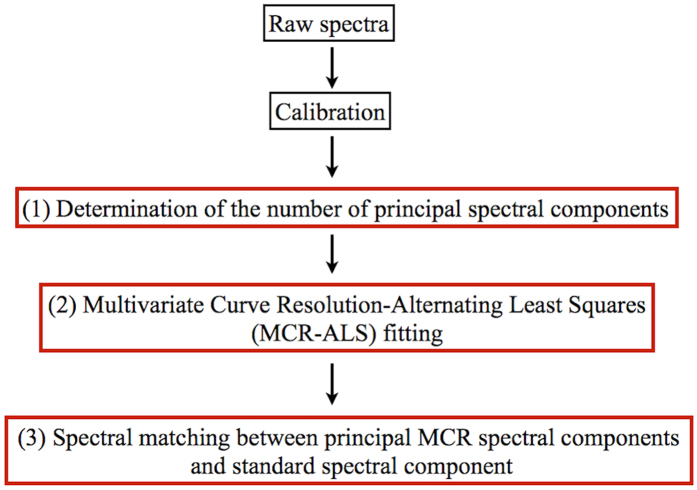
Flow chart of data analysis.

The flow chart of data analysis is shown in Fig. 6. Wavenumber calibration based on the standard spectrum of indene was carried out prior to the analysis. The analysis consists of the following three

steps: (1) determination of the number of principal spectral components contained in the observed spectra, (2) multivariate curve resolution-alternating least squares (MCR-ALS) fitting to decompose the observed spectra into spectrally interpretable components, (3) spectral matching between principal MCR spectral components and the standard spectrum.

#### (1) Determination of the number of principal spectral components

To determine the number of principal spectral components in the observed spectra, we introduced a new protocol based on signal-to-noise ratio (S/N) consideration. First, SVD analysis was performed to obtain SVD-decomposed spectra, *Intensity*^*SVDoriginal*^. Then, these SVD-decomposed spectra were smoothen by Savitzky-Golay (polynomial) method to obtain *Intensity*^*SVDsmooth*^, which was regarded as the “*Signal*”. Then, for each SVD-decomposed spectrum, *Intensity*^*SVDoriginal*^ can be written as,


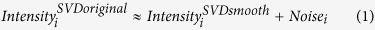


**Figure 7:**
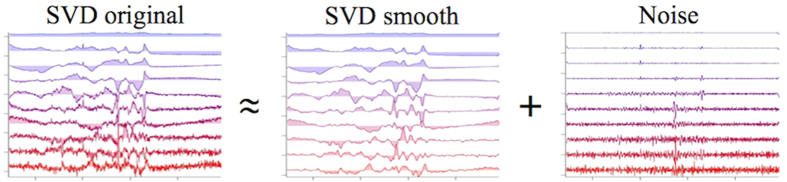
Signal and noise in SVD components.

Equation 1 can be visualized as shown Fig. 7.

The signal-to-noise ratio is defined as,


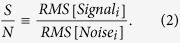


By using this method, we can automatically select out spectral components that have S/N ratios higher than a prefixed threshold value. In the present study, we fixed the threshold at 4. SVD spectra with S/N ratios higher than 4 were included in the subsequent analysis.

#### (2) Multivariate Curve Resolution - Alternating Least Squares (MCR-ALS)

A raw Raman spectrum is a superposition of a number of independent spectral components from different molecules existing in the tissue. Multivariate Curve Resolution-Alternating Least Squares (MCR-ALS) decomposes a large spectral data set into spectrally interpretable components. The experimental raw spectral data set *A* can be written as an *m* × *n* matrix, where *m* is the number of data points in one spectrum and *n* is the number of spectra in the data set; each column vector of *A, A*_*i*_ = (A_*1i*_, …, A_*mi*_)^T^, represents the *i-th* Raman spectrum having *m* data points. We decompose *A* into a product of two matrices *W* and *H*,


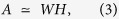


where *W* is an *m* × *k* matrix and *H* is an *k* × *n* matrix, *k* is the number of components determined by the S/N consideration in the first step. Equation (3) can be written in matrix form as,


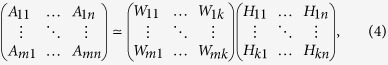


In equation (4), MCR-ALS decomposes the raw data set matrix *A* into the major spectral component matrix *W* and their spatial distribution matrix *H*. Each column vector of *W, W*_*i*_ =  (W_*1i*_, …, W_*mi*_) ^T^ is the *i-th* major spectral component and each row vector of *H, H*_*i*_ =  (H_*i1*_, H_*i2*_, H_*i3*_, H_*i4*_, …, H_*in*_) represents the intensity profile corresponding to *W*_*i*_.

During the process of the MCR analysis, *W* and *H* matrices are forced to be non-negative; i.e., *W* ≥ 0, *H* ≥ 0. The final solutions are obtained by iterative refinement to minimize the Frobenius norm ||*A-WH*||^2^. The SVD spectral components are used as initial guesses of the spectral components in the iteration process. The negative values in the SVD spectra are truncated to be zero. The present MCR-ALS analysis does not require orthogonality among column vectors in *W* and row vectors in *H*. Note that the component spectra and the intensity patterns are definitely not orthogonal to one another; in contrast, they are assumed to be orthogonal in other spectral decomposition methods like PCA and SVD. The details of the MCR-ALS method are given elsewhere^20^,^31^.

#### (3) Spectral matching between principal MCR spectral components and standard spectral component

The decomposed spectra *W*_*1*_, …, *W*_*k*_ and the standard spectrum *S*_*std*_ were normalized so that their norms are unity. These normalized vectors can be written as,


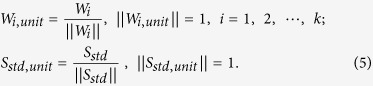


Then, we calculate the similarity Unit Normalized Euclidean Distance (UNED) between *W*_*i,unit*_ and *S*_*std,unit*_ as follows,


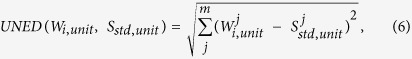


where *j* is the index of the *j-th* element of the m-dimensional vectors *W*_*i,unit*_ and *S*_*std,unit*_.

**Figure 8:**
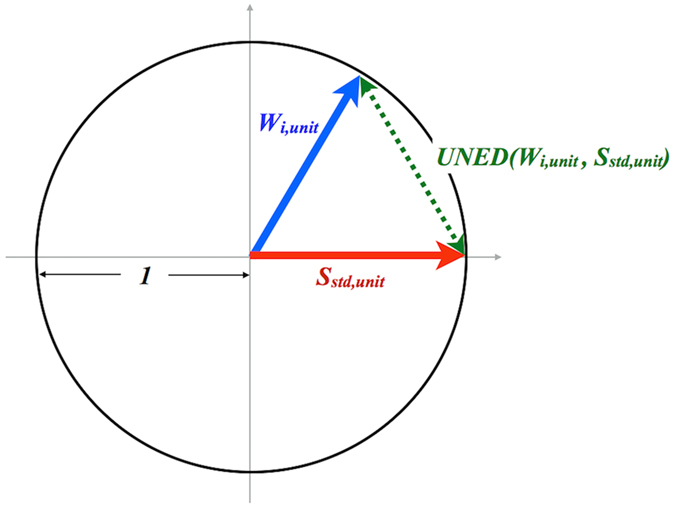
A schematic 2-dimensional model showing the principle of the UNED analysis.

Figure 8 schematically shows the principle of the UNED analysis, represented in 2-D space for simplicity. UNED represents the distance between a normalized MCR-decomposed spectral component and the normalized standard spectrum, whose minimum value is 0 (two identical vectors) and maximum value is 2 (two vectors along opposite direction). Therefore, the smaller the UNED value is, the larger is the similarity. Thus, from UNED, we can evaluate the distance between the two normalized spectral vectors to know how similar the two spectra are.

## Additional Information

**How to cite this article**: Chen, P.-H. *et al*. Automatic and objective oral cancer diagnosis by Raman spectroscopic detection of keratin with multivariate curve resolution analysis. *Sci. Rep.* **6**, 20097; doi: 10.1038/srep20097 (2016).

## References

[b1] Sun T.-T., Shih C. & Green H. Keratin cytoskeletons in epithelial cells of internal organs. Proc. Natl. Acad. Sci. USA 76, 2813–2817 (1979). 111242 10.1073/pnas.76.6.2813 PMC383699

[b2] Sivaramakrishnan S., DeGiulio J. V., Lorand L., Goldman R. D. & Ridge K. M. Micromechanical properties of keratin intermediate filament networks. Proc. Natl. Acad. Sci. USA 105, 889–894 (2008). 18199836 10.1073/pnas.0710728105 PMC2242724

[b3] Schlegel R., Banks-Schlegel S., McLeod J. A. & Pinkus G. S. Immunoperoxidase Localization of Keratin in Human Neoplasms. Am. J. Pathol. 101, 41–49 (1980). 6160769 PMC1903585

[b4] Ramaekers F. C. S. . Use of antibodies to intermediate filaments in the characterization of human tumors. Cold Spring Harb. Symp. Quant. Biol. 46, 331–339 (1982). 6179695 10.1101/sqb.1982.046.01.034

[b5] Mall R., Franke W. W. & Schiller D. L. The catalog of human cytokeratins: patterns of expression in normal epithelia, tumors and cultured cells. Cell 31, 11–24 (1982). 6186379 10.1016/0092-8674(82)90400-7

[b6] Trask D. K. . Keratins as markers that distinguish normal and tumor-derived mammary epithelial cells. J. Cell Biol. 87, 2319–2323 (1990). 10.1073/pnas.87.6.2319 PMC53678 1690428

[b7] Ogden G. R., Lane E. B., Hopwood D. V. & Chisholm D. M. Evidence for field change in oral cancer based on cytokeratin expression. Br. J. Cancer 67, 1324–1330 (1993). 7685618 10.1038/bjc.1993.245 PMC1968511

[b8] Choontharu M. M., Binda A., Bhat B. & Mahalinga S. Role of tumor markers in oral squamous cell carcinoma: Review of literature and future consideration. SRM Journal of Research in Dental Sciences 3, 251–256 (2012).

[b9] Venkatakrishna K. . Optical pathology of oral tissue: A Raman spectroscopy diagnostic method. Curr. Sci. 80, 665–669 (2001).

[b10] Krishna C. M. . Micro-Raman spectroscopy for optical pathology of oral squamous cell carcinoma. Appl. Spectrosc. 58, 1128–1135 (2004). 15479531 10.1366/0003702041959460

[b11] Malini R. . Discrimination of normal, inflammatory, premalignant, and malignant oral tissue: a Raman spectroscopy study. Biopolymers 81, 179–193 (2006). 16231284 10.1002/bip.20398

[b12] Guze K. . Parameters defining the potential applicability of Raman spectroscopy as a diagnostic tool for oral disease. J. Biomed. Opt. 14, 014016-1-014016-9 (2009). 10.1117/1.3076195 19256704

[b13] Guze K., Short M., Zeng H., Lerman M. & Sonis S. Comparison of molecular images as defined by Raman spectra between normal mucosa and squamous cell carcinoma in the oral cavity. J. Raman Spectrosc. 42, 1232–1239 (2011).

[b14] Krafft C. . Advances in optical biopsy – correlation of malignancy and cell density of primary brain tumors using Raman microspectroscopic imaging. Analyst 137, 5533–5537 (2012). 23050263 10.1039/c2an36083g

[b15] Bergner N. . Unsupervised unmixing of Raman microspectroscopic images for morphochemical analysis of non-dried brain tumor specimens. Anal. Bioanal. Chem. 403, 719–725 (2012). 22367289 10.1007/s00216-012-5858-1

[b16] Singh S. P., Deshmukh A., Chaturvedi P. & Krishna C. M. *In vivo* Raman spectroscopic identification of premalignant lesions in oral buccal mucosa. J. Biomed. Opt. 17, 105002-1-105002-9 (2012). 10.1117/1.JBO.17.10.105002 23223996

[b17] Singh S. P., Sahu A., Deshmukh A., Chaturvedi P. & Krishna C. M. *In vivo* Raman spectroscopy of oral buccal mucosa: a study on malignancy associated changes (MAC)/cancer field effects (CFE). Analyst 138, 4175–4182 (2013). 23392131 10.1039/c3an36761d

[b18] Rekha P. . Raman spectroscopic characterization of blood plasma of oral cancer. IEEE ICP 135–137 (2013).

[b19] Guze K. . Pilot study: Raman spectroscopy in differentiating premalignant and malignant oral lesions from normal mucosa and benign lesions in humans. Head & Neck 37, 511–517 (2015). 24677300 10.1002/hed.23629

[b20] Juan A. D., Jaumot J. & Tauler R. Multivariate Curve Resolution (MCR). Solving the mixture analysis problem, Anal. Methods 6, 4964–4976 (2014).

[b21] Lee D.-D. & Seung H.-S. Learning the parts of objects by non-negative matrix factorization Nature 401, 788–791 (1999). 10548103 10.1038/44565

[b22] Spiro T. G. & Strekas T. C. Resonance Raman Spectra of Heme Proteins. Effects of Oxidation and Spin State. J. Am. Chem. Soc. 96, 338–345 (1974). 4361043 10.1021/ja00809a004

[b23] Robila S. A. & Gershman A. Spectral Matching Accuracy in Processing Hyperspectral Data. IEEE ISSCS 1, 163–166 (2005).

[b24] Chang C.-I. Spectral information divergence for hyperspectral image analysis. IEEE IGARSS 1, 509–511 (1999).

[b25] Du Y. . New hyperspectral discrimination measure for spectral characterization. Opt. Eng. 43, 1777–1786 (2004).

[b26] Moll R., Krepler R. & Franke W. W. Complex Cytokeratin Polypeptide Patterns Observed in Certain Human Carcinomas. Differentiation 23, 256–269 (1983). 6189757 10.1111/j.1432-0436.1982.tb01291.x

[b27] Fuchs E., Grace M. P., Kim K. H. & Marchuk D. Differential expression of two classes of keratins in normal and malignant epithelial cells and their evolutionary conservation. Cancer Cells 1: The Transformed Phenotype, eds Levine A., Topp W., Vande Woude G., Watson J. D. (Cold Spring Harbor Laboratory, New York), pp 161–167 (1984).

[b28] Steinert P. M. & Freedberg I. M. Molecular and cellular biology of keratins. The Physiology, Biochemistry and Molecular Biology of the Skin, eds Goldsmith L. A. (Oxford University Press, New York), pp 113–147 (1991).

[b29] Kikuchi S., Aosaki T., Bito K., Naito S. & Katayama Y. *In vivo* evaluation of lateral lipid chain packing in human stratum corneum. Skin Res. Technol. 21, 76–83 (2015). 24889490 10.1111/srt.12159

[b30] Hamaguchi H. Calibrating Multichannel Raman Spectrometers. Appl. Spectrosc. Rev. 24, 137–174 (1988).

[b31] Ando M. & Hamaguchi H. Molecular component distribution imaging of living cells by multivariate curve resolution analysis of space resolved Raman spectra. J. Biomed. Opt. 19, 011016-1-011016-6 (2014). 10.1117/1.JBO.19.1.011016 24108582

